# Novel endoscopic management for acute diverticulitis with localized abscess

**DOI:** 10.1055/a-2325-2694

**Published:** 2024-06-05

**Authors:** Hai-Bin Zhang, Ben-Song Duan, Jia-Ning Shi, Yuan Chu, Cheng Guo

**Affiliations:** 1 Endoscopy Center, Department of Gastroenterology, Shanghai East Hospital, Tongji University School of Medicine, Shanghai, China

The clinical spectrum of acute diverticulitis ranges from a phlegmon to limited abscesses, to free perforation with purulent or contaminated peritonitis 1 . While there is little debate about the optimal treatment for mild or very severe situations, uncertainty remains about the optimal strategy for acute diverticulitis with localized abscesses. Here, we report a successful endoscopic diverticulotomy for limited septic diverticulitis caused by a fecal stone.

A 44-year-old man experienced sudden abdominal pain 1 week earlier and computed tomography scan at a local hospital showed a high density shadow in the colon ( Fig. 1 **a** ). Colonoscopy showed a mucosal defect in the ascending colon ( Fig. 1 **b** ). However, after 3 days of antibiotic treatment, the patient’s abdominal pain worsened. Blood tests showed a threefold increase in C-reaction protein to 36 mg/L and a twofold increase in white blood cell counts to 18 × 10 ^9^ /L. The patient was then referred to our endoscopy center and underwent colonoscopy.

**Fig. 1:**
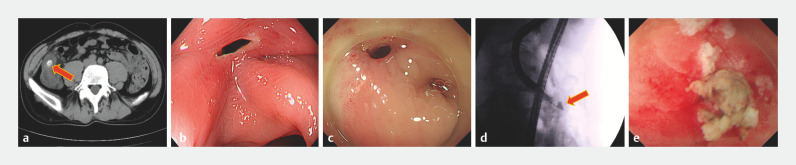
Limited septic diverticulitis caused by an embedded fecal stone. **a** Computed tomography showed a high density shadow (arrow) in the colon. **b** A mucosal defect was seen in the ascending colon. **c** The diverticular opening was congested and edematous. **d** X-ray showed an approximately 1-cm diverticulum (arrow). **e** Photograph of the yellow fecal stone.

Septic diverticulitis was considered first. The diverticular opening was congested and edematous ( Fig. 1 **c** ), and white pus could be drawn. X-ray showed an approximately 1-cm diverticulum with inflammatory exudates, fortunately without perforation ( Fig. 1 **d** ). Inspired by endoscopic septum division for esophageal diverticulum 2 , the diverticular septum was incised carefully and a yellow fecal stone, about 0.6 cm in diameter, slipped out ( Fig. 1 **e** , Video 1 ). The bottom and the opening of the diverticulum were treated with electrocoagulation and closed by endoloop-assisted clip closure. Following this treatment and 3 days of antibiotic therapy, the patient’s abdominal pain resolved and blood test results returned to the normal range.

Endoscopic diverticulotomy with stone extraction for limited septic diverticulitis.Video 1

This patient was diagnosed with a diverticular abscess caused by an embedded fecal stone in the diverticulum. Antibiotic therapy alone for septic diverticulitis is accompanied by a high risk of recurrence owing to the persistence of the etiology 3 . In this case, we endoscopically removed the fecal stone, drained the pus, destroyed the diverticulum, and sutured with endoloop-assisted clip closure, thus avoiding the need for surgery. During a 1 year follow-up period, the patient had no further acute diverticulitis. This case demonstrates novel endoscopic management for acute diverticulitis with localized abscesses.

Endoscopy_UCTN_Code_TTT_1AQ_2AJ

## Notes

Endoscopy E-Videos https://eref.thieme.de/e-videos
*E-Videos* is an open access online section of the journal *Endoscopy* , reporting on interesting cases and new techniques in gastroenterological endoscopy. All papers include a high-quality video and are published with a Creative Commons CC-BY license. Endoscopy E-Videos qualify for HINARI discounts and waivers and eligibility is automatically checked during the submission process. We grant 100% waivers to articles whose corresponding authors are based in Group A countries and 50% waivers to those who are based in Group B countries as classified by Research4Life (see: https://www.research4life.org/access/eligibility/ ). This section has its own submission website at https://mc.manuscriptcentral.com/e-videos .
